# Effect of Assisted Surgery on Work-Related Musculoskeletal Disorder Prevalence by Body Area among Surgeons: Systematic Review and Meta-Analysis

**DOI:** 10.3390/ijerph20146419

**Published:** 2023-07-20

**Authors:** Philippe Gorce, Julien Jacquier-Bret

**Affiliations:** 1 International Institute of Biomechanics and Occupational Ergonomics, ErBio, Avenue du Dr Marcel Armanet, 83400 Hyères, France; gorce@univ-tln.fr; 2 Université de Toulon, CS60584, CEDEX 9, 83041 Toulon, France

**Keywords:** musculoskeletal disorders, prevalence, body area, surgeons, assisted surgery, systematic review, meta-analysis, worldwide analysis

## Abstract

Surgeons are highly exposed to work-related musculoskeletal disorders (WMSDs). The objective of this review was to summarize the WMSD prevalence by body area with and without assistive devices. The underlying question was whether there is an effect of assistive device use (robot, video, or other) during surgery on WMSD prevalence by body area among surgeons, regardless of their specialty. The systematic review was conducted according to the PRISMA guidelines. The Google Scholar, Pubmed/Medline, and ScienceDirect databases were scanned to identify relevant studies. The article selection, review, critical appraisal, and data extraction were performed by two authors independently. Among the 34,854 unique identified records, 77 studies were included. They were divided into two groups: 35 focused on robotic- and video-assisted surgery (RVAS) and 48 concerning surgery without video/robotic assistance (WAS) (6 studies evaluated the prevalence for both groups). WMSD prevalence was reported for 13 body areas: the neck, back, upper back, mid-back, lower back, shoulders, elbows, wrists, fingers, thumbs, hips, knees, and ankles. The results showed that WMSD prevalence was significantly higher (unpaired *t*-test, *p* < 0.05) for RVAS in the shoulders (WAS: 28.3% vs. RVAS: 41.9%), wrists (WAS: 20.9% vs. RVAS: 31.5%), and thumbs (WAS: 9.9% vs. RVAS: 21.8%). A meta-analysis was performed for 10 body areas (with 4 areas including more than 25 studies). No sufficient data were available for the mid-back, thumbs, or hips. A high heterogeneity (Cochran’s Q test and I^2^ statistic) was observed. A random-effects model revealed that the highest worldwide prevalence was in the neck (WAS: 41% and RVAS: 45.3%), back (WAS: 37.7% and RVAS: 49.9%), lower back (WAS: 40.0% and RVAS: 37.8%), and shoulders (WAS: 27.3% and RVAS: 41.4%). Future work could focus on work environment design, particularly the positioning and adjustment of equipment, and on postural analysis to reduce the appearance of WMSDs. Recommendations are proposed for future reviews and meta-analyses.

## 1. Introduction

Surgeons are confronted with significant risks of work-related musculoskeletal disorders (WMSDs). Their overall prevalence has been estimated at 83% by Szeto et al. [1] and 90% by Liang et al. [2]. The high risk of WMSDs is directly related to the practice of surgeons, which requires a high level of mental concentration [3], a high physical load with a high precision level [4], and repeated and prolonged awkward static postures [5]. Physical and psychological loads induce fatigue that can lead to a decrease in productivity and the quality of work [6].

Understanding the mechanisms that lead to WMSDs requires knowledge of the most susceptible body areas. A large number of studies have reported prevalence in the neck, lower back, shoulders, and wrists. Plerhoples et al. [7] and Khansa et al. [8] quantified a prevalence of 46.6% and 66.6% in the neck among 1068 and 865 surgeons, respectively. From 736 and 223 surgeons, Wolhauer et al. [9] and Mohseni-Bandpei et al. [10] indicated a prevalence of 39.0% and 71.7%, respectively, for the lower back. For the shoulders, Ruitenburg et al. [11] found a prevalence of 25.8% (295 surgeons), and Liang et al. [2] observed a prevalence of 61.5% (354 surgeons). From a sample of 578 and 865 surgeons, Alqhatani et al. [12] and Khansa et al. [8] reported a prevalence of 31.3% and 38.3%, respectively, for the wrists. Other less-studied areas, such as the upper back (58.4% [13]) and the lower limbs (hips: 28.5%, knees: 48.7%, and ankles: 27.9% [14]), also present a significant prevalence. These WMSDs expose surgeons to numerous pathologies, such as spinal degeneration or carpal tunnel syndrome [15], which can lead them to take medication, consult physiotherapists, or undergo surgery [10].

The diversity of operations that surgeons perform requires a wide range of skills. Surgeons traditionally operate in open surgery. Progressively, minimally invasive techniques have been developed. They have been reinforced by assistive devices such as robots or videos, with the technological progress in the service of the patients. Several studies have suggested that robotic surgery and assistive devices are more ergonomically favorable and potentially less stressful than conventional surgery [16,17]. However, the literature evaluating the ergonomic benefits remains sparse. Robotic surgery has been shown to be associated with high levels of strain [18]. Park et al. [19] and Franasiak et al. [20] reported a fatigue effect when performing minimally invasive surgery. The Society of American Gastrointestinal Endoscopic Surgeons Task Force on Ergonomics showed that 8 to 12% of surgeons had neck and upper-extremity pain during laparoscopic surgery [21]. Studies in assisted surgery have described a high prevalence in the most exposed areas, such as the neck (58.8% among 260 surgeons [20] and 72.9% among 495 surgeons [22]), lower back (44.2% among 736 surgeons [9] and 68.1% among 135 surgeons [1]), shoulders (77% [23] and 51.2% [24] among 284 and 285 surgeons, respectively), and wrists (44.2% among 582 surgeons [25] and 60.9% among 495 surgeons [22]). 

However, there are no studies that have directly evaluated the effect of the use of technical assistance on the risk of WMSDs in surgeons by body area. To the best of our knowledge, the only meta-analysis proposed in the literature is the work of Epstein et al. [15]. The authors evaluated the occurrence of common pathologies suffered by surgeons in a pool of 21 studies. Their meta-analysis reported the prevalence for only four body areas. Generalizing this analysis to all body areas would provide a better understanding of the WMSD risks to which surgeons are exposed. The consideration of the presence or absence of assistance would allow us to provide some answers as to the ergonomic contributions of assistive devices to the risks of WMSDs for surgeons.

The objective of this work was to study the effect of the use of assistive devices (robot, video, or other) on the WMSD prevalence in each body area.

## 2. Materials and Methods

### 2.1. Search Strategy and Study Eligibility

The systematic search was conducted in the ScienceDirect, PubMed/Medline, and Google Scholar databases without any restriction on the publication date between July and October 2022. The preferred reporting items for systematic reviews and meta-analyses (PRISMA) guidelines were used [26]. The protocol for this review was registered at PROSPERO (CRD42023415342). The search focused on studies that evaluated the WMSD prevalence by body area among surgeons, regardless of their specialty. The following keywords were used: “musculoskeletal disorders” AND “surgeon” AND “prevalence” AND “body area”. 

Studies were included in the review if they (1) reported data on the MSD prevalence in surgeons and (2) were research articles (regardless of the study design, i.e., experimental protocol, cross-sectional studies, etc.) published in English. Studies were excluded if they (1) were not published in English; (2) were reviews, systematic reviews, conferences, commentaries, case studies, or case series; (3) were not about surgeons; or (4) did not provide detailed MSD prevalence information by body area.

All entries were compiled from the three databases into a unique table to remove duplicates. Then, two reviewers (PG and JJB) worked together to progressively reduce the list of studies by applying successive conditional formatting and personalized filters based on inclusion criteria, retaining only those articles corresponding to the objective. From the remaining list, the two reviewers separately screened the studies and assessed content according to the inclusion/exclusion criteria for final selection. Finally, they separately performed the critical appraisal and extracted the data from included studies. The data were reported in an overview table by each reviewer and summarized in a final table. In the case of a disagreement, a final decision was obtained by consensus after re-reviewing the article.

### 2.2. Quality Assessment and Data Extraction

The extracted data included the country, number of participants, male and female repartition, the surgeons’ ages, the case load per week, the surgeons’ number of years in practice, whether the surgeries were robotic-/video-assisted or not, and the WMSD prevalence by body area. The main outcome was the prevalence for thirteen body areas: the neck, back, upper back, mid-back, lower back, shoulders, elbows, wrists, fingers, thumbs, hips, knees, and ankles. The relative data were reported in a summary table. Where data were missing, the box was left blank. For the prevalence, if the information covered the entire limb (e.g., the lower limb), the data were not retained. Effect measures were reported with prevalence if available. The studies were divided into two groups: robotic-assisted and video-assisted surgery (RVAS) vs. surgery without video/robotic assistance (WAS). 

The modified CONSORT 2010 checklist [27] was used to assess the quality of the included articles. The quality level was obtained using the McFarland and Fischer classification [28]: high (≥85% of the checklist items), medium (50 to 85% of the checklist items), or low (<50% of the checklist items).

### 2.3. Statistical Analysis

The effect of assistance during surgery was evaluated on each mean prevalence by body area using an independent sample *t*-test. Parametric *t*-tests were chosen if the distribution was normal (Shapiro–Wilk test). Otherwise, a non-parametric *t*-test was performed.

A meta-analysis was performed based on the work of Neyeloff et al. [29] on body areas where the number of studies was higher than five. The heterogeneity of the studies was assessed using Cochran’s Q test (significance level < 10%) and the I^2^ statistic (significance level > 50%). In the case of heterogeneity, a random-effects model with an inverse-variance approach was employed. Otherwise, a fixed-effects model was applied.

## 3. Results

### 3.1. Search Results

**Figure 1:**
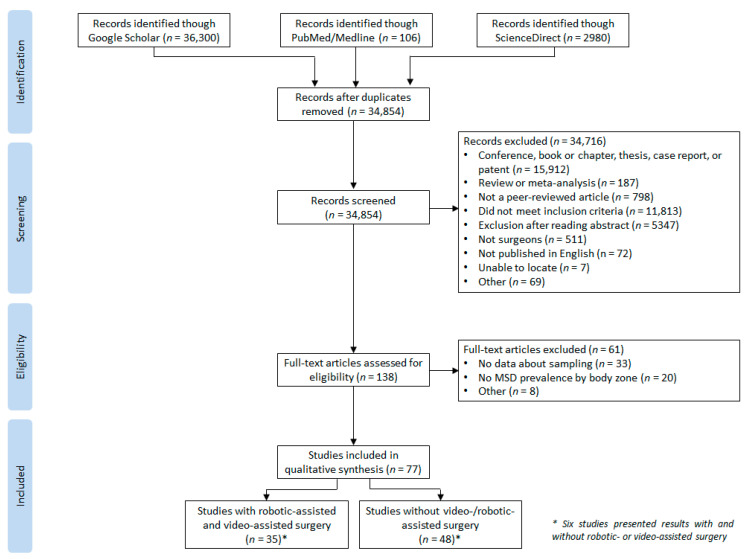
PRISMA flow chart.

The searches returned 34,854 unique records after the duplicates were deleted. Then, 34,716 were excluded from the title/abstract screening. The 138 remaining articles were fully assessed. Sixty-one were discarded, mainly due to the absence of WMSD prevalence by body areas and insufficient data about the sampling. Ultimately, 77 studies were included in the present review. The search process is shown in Figure 1.

### 3.2. Quality Appraisal

The studies by Giagio et al. [30] and Mohseni-Bandpei et al. [10] were assessed as being of high quality. The other 75 works were evaluated as being of medium quality (number of items present was between 50 and 85%; see Table 1 and Appendix A.1 for a detailed assessment). 

### 3.3. Study Characteristics

All included articles were surveys that were designed to assess the WMSD risk of surgeons. Among the 77 included studies, 17 specialties were identified (cardiology, dentistry, dermatology, gastroenterology, general surgery, hand surgery, neurology, gynecology, oncology, ophthalmology, orthopedics, otorhinolaryngology, pediatrics, plastic surgery, thoracic surgery, urology, and vascular surgery). The subjects generally included both men and women. Two studies included only men [55,75], one included only women [42], and sixteen studies did not report this information. The average participant’s age was 30 to 54 years, and they had an overall experience of 3 to 22 years and a mean case load of 1.9 to 33.8 h or 0.7 to 24.7 cases per week. The samples in the different studies were heterogeneous, ranging from 17 [50,54] to 1086 surgeons [7]. The selected studies covered a wide range of countries from different continents. 

The studies were divided into two groups: surgery without video/robotic assistance (WAS) and robotic-assisted and video-assisted surgery (RVAS). The general population characteristics, i.e., the country, the number of participants, the male/female repartition, the mean age, the mean practice and mean case load per week, and the prevalence of WMSDs by body area of the 77 included studies, were summarized. Thirteen areas were identified. The neck, shoulders, and wrists were the most investigated areas, with 65, 61, and 58 studies, respectively. The thumbs, mid-back, fingers, and hips were the least evaluated areas, with 4, 6, 11, and 13 studies, respectively. The ankle, knee, back, upper back, elbow, and lower back prevalence was reported in 22, 26, 26, 30, 35, and 44 studies, respectively. No data about the effect estimate or summary statistics were considered because they were not included in the various works. The summarized data are detailed in Appendix A.2 (WAS studies) and Appendix A.3 (RVAS studies).

### 3.4. Meta-Analysis of WMSD Prevalence by Body Area

A meta-analysis was performed on the different body areas studied in order to investigate their prevalence for assisted and unassisted surgery. Thirteen areas were included in the analysis. However, the amount of data available in the included works did not allow for an analysis of the prevalence in the thumbs, mid-back, and hips for robotic-assisted and video-assisted surgery. The meta-analysis was, therefore, conducted on the 10 remaining body areas.

The heterogeneity among the studies was assessed using the Q and I^2^ statistics. The results revealed important heterogeneity for these ten body areas. The results for WAS were as follows: neck, Q = 1684.3, df = 38, I^2^ = 97.7%, and *p* < 0.001; back, Q = 211.6, df = 12, I^2^ = 94.3%, and *p* < 0.001; upper back, Q = 858.1, df = 20, I^2^ = 97.6%, and *p* < 0.001; lower back, Q = 885.1, df = 32, I^2^ = 96.3%, and *p* < 0.001; shoulders, Q = 1103.5, df = 35, I^2^ = 96.8%, and *p* < 0.001; elbows, Q = 384.1, df = 23, I^2^ = 94.0%, and *p* < 0.001; wrists, Q = 871.7, df = 36, I^2^ = 95.8%, and *p* < 0.001; fingers, Q = 124.6, df = 4, I^2^ = 96.7%, and *p* < 0.001; knees, Q = 378.8, df = 19, I^2^ = 94.9%, and *p* < 0.001; and ankles, Q = 372.6, df = 19, I^2^ = 94.9%, and *p* < 0.001. For RVAS, the results were as follows: neck, Q = 1038.2, df = 31, I^2^ = 97.0%, and *p* < 0.001; back, Q = 789.5, df = 16, I^2^ = 97.9%, and *p* < 0.001; upper back, Q = 248.8, df = 10, I^2^ = 95.9%, and *p* < 0.001; lower back, Q = 393.6, df = 12, I^2^ = 96.9%, and *p* < 0.001; shoulders, Q = 642.6, df = 29, I^2^ = 95.4%, and *p* < 0.001; elbows, Q = 76.2, df = 13, I^2^ = 82.9%, and *p* < 0.001; wrists, Q = 705.9, df = 26, I^2^ = 96.3%, and *p* < 0.001; fingers, Q = 66.1, df = 8, I^2^ = 87.9%, and *p* < 0.001; knees, Q = 68.2, df = 8, I^2^ = 88.2%, and *p* < 0.001; and ankles, Q = 88.8, df = 4, I^2^ = 95.4%, and *p* < 0.001. Considering the important heterogeneity among the results, a random-effects model was applied to evaluate the MSD prevalence in different body areas.

#### 3.4.1. Neck

**Figure 2:**
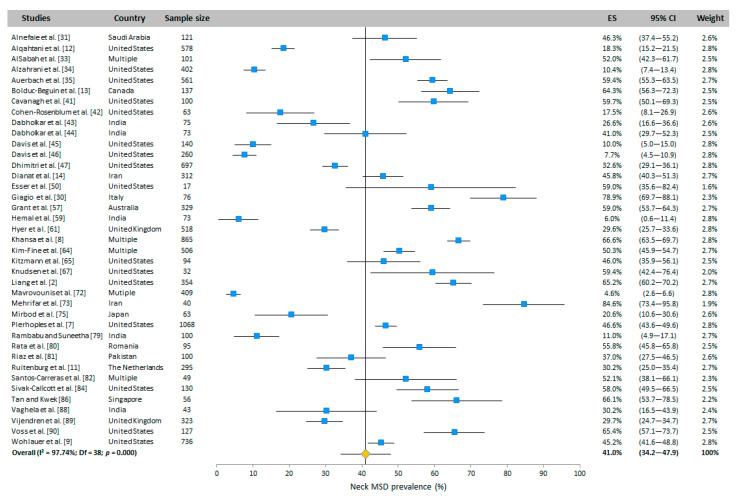

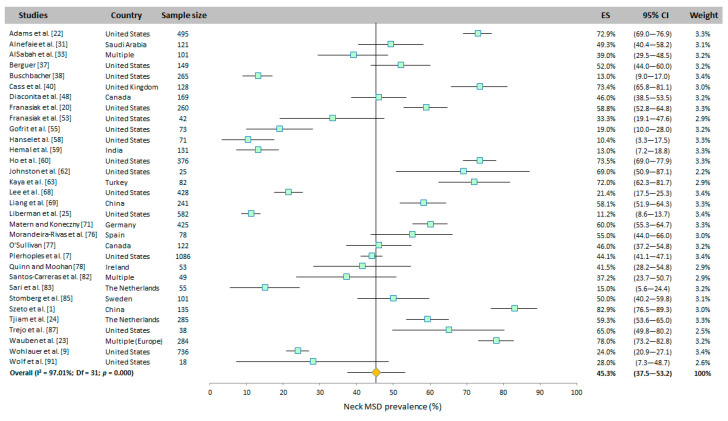
Meta-analysis of neck WMSD prevalence for WAS (top panel in blue) and for RVAS (bottom panel in green). The orange diamond represents the overall prevalence for each group.

The WMSD prevalence in the neck was presented in most of the included studies (39 WAS studies vs. 32 RVAS studies) that were carried out in many countries of the world (Figure 2). Based on the random-effects model, the neck prevalence was 41.0% (95% CI: 34.2–47.9%) and 45.3% (95% CI: 37.5–53.2%) for WAS and RVAS, respectively.

#### 3.4.2. Back

The back WMSD prevalence was evaluated in 13 WAS and 17 RVAS studies around the world. The overall prevalence was 37.7% (95% CI: 27.4–48.1%) and 49.6% (95% CI: 35.1–64.1%) for WAS and RVAS, respectively, obtained with the random-effects model (Appendix A.4).

#### 3.4.3. Upper Back

The prevalence of upper back WMSD was mentioned in 21 WAS and 11 RVAS studies around the world. The results of the random-effects model showed that the prevalence was 30.4% (95% CI: 22.6–38.2%) and 28.7% (95% CI: 19.1–38.2%) for WAS and RVAS, respectively (Appendix A.5).

#### 3.4.4. Lower Back

**Figure 3:**
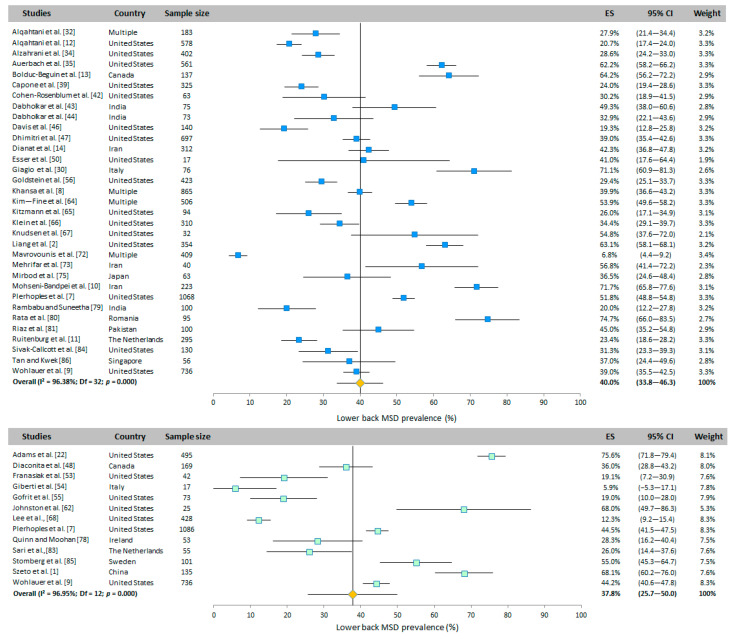
Meta-analysis of lower back WMSD prevalence for WAS (top panel in blue) and for RVAS (bottom panel in green). The orange diamond represents the overall prevalence for each group.

The prevalence of lower back WMSD is presented in Figure 3. This was assessed in 33 WAS and 13 RVAS studies from many countries. Based on the random-effects model results, the overall prevalence was 40.0% (95% CI: 33.8–46.3%) and 37.8% (95% CI: 25.7–50.0%) for WAS and RVAS, respectively.

#### 3.4.5. Shoulders

**Figure 4:**
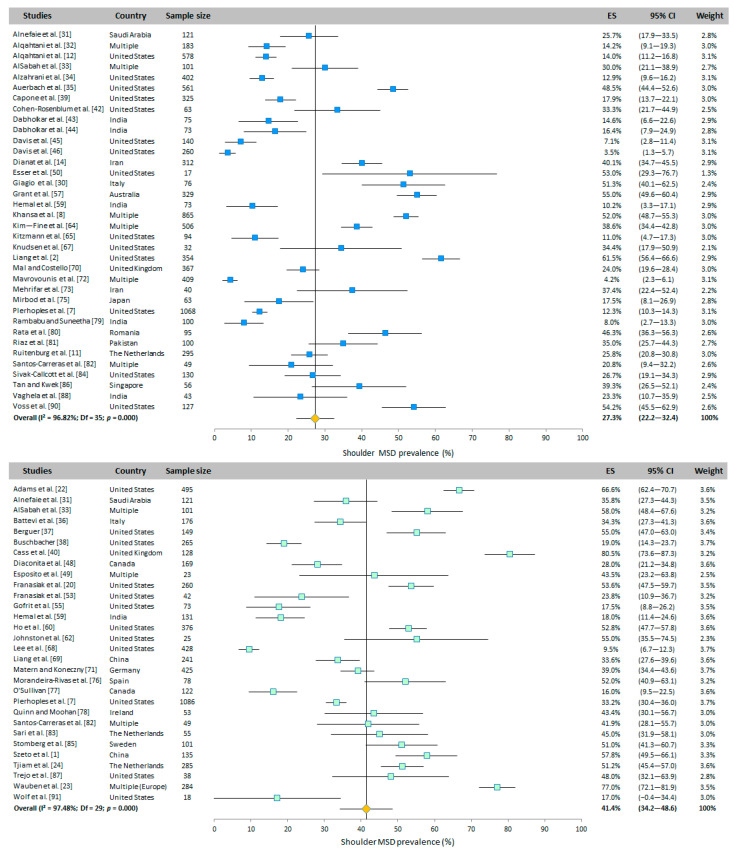
Meta-analysis of shoulder WMSD prevalence for WAS (top panel in blue) and for RVAS (bottom panel in green). The orange diamond represents the overall prevalence for each group.

The shoulder WMSD prevalence was evaluated in 36 WAS and 30 RVAS studies around the world. The overall prevalence was 27.3% (95% CI: 22.2–32.4%) and 41.4% (95% CI: 34.2–48.6%) for WAS and RVAS, respectively, obtained with the random-effects model (Figure 4).

#### 3.4.6. Elbows

**Figure 5:**
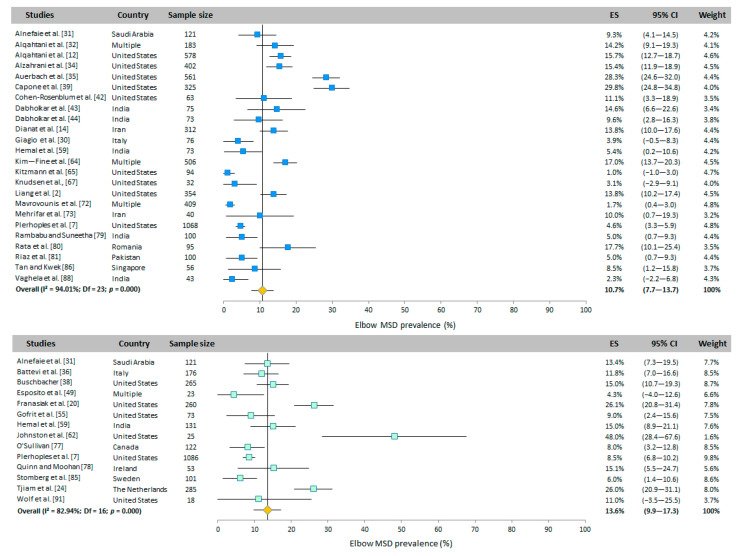
Meta-analysis of elbow WMSD prevalence for WAS (top panel in blue) and for RVAS (bottom panel in green). The orange diamond represents the overall prevalence for each group.

The prevalence of WMSD in the elbows was addressed in 24 studies for WAS and 17 studies for RVAS (Figure 5). Based on the results of the random-effects model, the elbow WMSD prevalence was 10.7% (95% CI: 7.7–13.7%) and 13.6% (95% CI: 9.9–17.3%) for WAS and RVAS, respectively.

#### 3.4.7. Wrists

**Figure 6:**
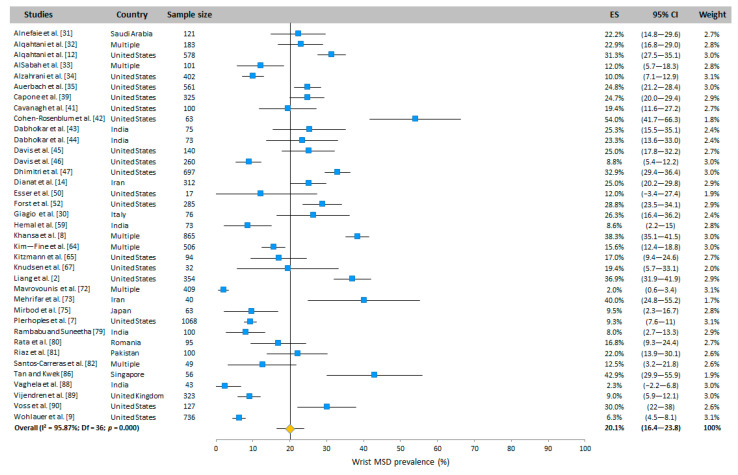

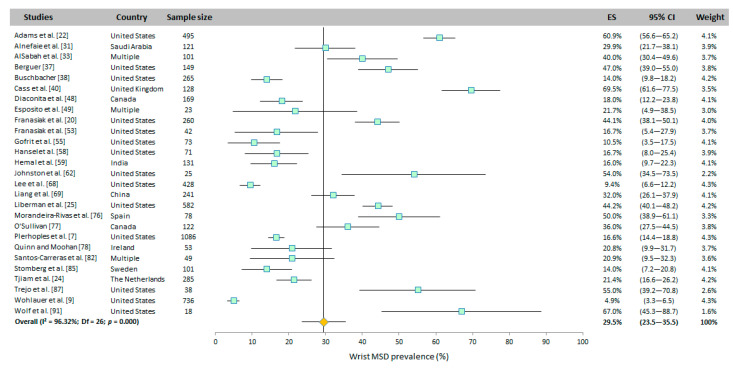
Meta-analysis of wrist WMSD prevalence for WAS (top panel in blue) and for RVAS (bottom panel in green). The orange diamond represents the overall prevalence for each group.

The prevalence of wrist WMSD is presented in Figure 6. This was assessed in 37 WAS and 27 RVAS studies performed in many countries. Based on the random-effects model results, the prevalence was 20.1% (95% CI: 16.4–23.8) and 29.5% (95% CI: 23.5–35.5) for WAS and RVAS, respectively.

#### 3.4.8. Fingers

The prevalence of MSD in the fingers was reported in five WAS and nine RVAS studies conducted all around the world. The results of the random-effects model indicated that the finger prevalence was 20.3% (95% CI: 8.8–31.8%) and 22.2% (95% CI: 15.6–28.7%) for WAS and RVAS, respectively (Appendix A.6).

#### 3.4.9. Knees

The knee WMSD prevalence was evaluated in twenty WAS and nine RVAS studies around the world. The overall prevalence was 15.5% (95% CI: 11.2–19.8%) and 18.5% (95% CI: 12.9–24.2%) for WAS and RVAS, respectively, obtained with the random-effects model (Appendix A.7).

#### 3.4.10. Ankles

The prevalence of ankle WMSD is detailed in Appendix A.8. This was assessed in twenty WAS and five RVAS studies performed in many countries. Based on the results of the random-effects model, the prevalence was 13.9% (95% CI: 10.5–17.4%) and 15.2% (95% CI: 7.5–22.9%) for WAS and RVAS, respectively.

### 3.5. Body Area WMSD Prevalence

**Figure 7:**
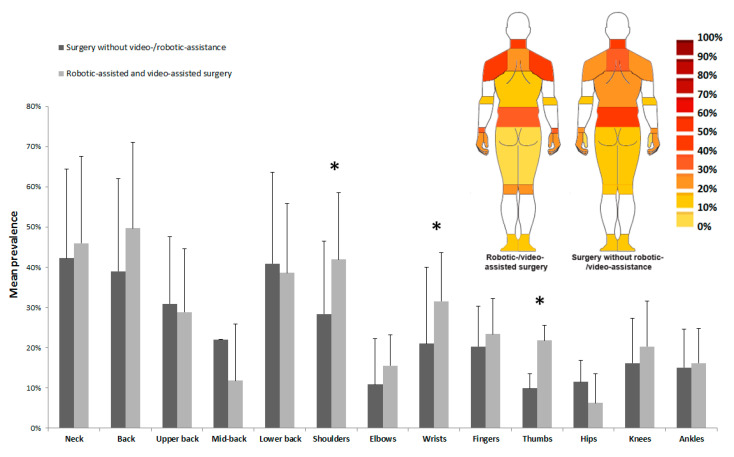
WMSD mean prevalence by body area for the two groups of studies. Vertical bars represent the standard deviation. * indicates a significant difference (*p* < 0.05) in the prevalence between the two groups (with and without robotic-/video-assisted surgery). Body maps of the WMSD prevalence by body areas with and without robotic-/video-assisted surgery are presented in the upper right corner.

The prevalence by body area as well as the effect of assistance during surgery are represented in Figure 7. The neck (42.3 ± 21.5% vs. 46.0 ± 22.1%), back (39.0 ± 21.4% vs. 49.7 ± 23.0%), lower back (40.9 ± 17.2% vs. 38.6 ± 22.8%), and shoulders (28.3 ± 16.7% vs. 41.9 ± 18.2%) were the most exposed areas to WMSDs in WAS and RVAS, respectively. An effect of assistance was observed for the shoulders, wrists, and thumbs. For these three areas, surgery with assistance increased the WMSD prevalence by at least 10%. Figure 7 also displays a body map of the WMSD prevalence by body area for WAS and RVAS. Statistical analyses using *t*-tests revealed the effect of assistive equipment use on the WMSD prevalence in these three body areas. For the shoulders (WAS: 28.3% vs. RVAS: 41.9%), wrists (WAS: 20.9% vs. RVAS: 31.5%), and thumbs (WAS: 9.9% vs. RVAS: 21.8%), the prevalence was higher in RVAS (*p* < 0.05).

## 4. Discussion

The aim of this study was to propose a literature review and meta-analysis to investigate the prevalence of WMSD among surgeons. The objective was to analyze the effect of robotic/video assistance during surgery on the worldwide WMSD prevalence by body area. Seventy-seven studies were included in the analysis. Thirty-five studies investigated robotic-assisted and video-assisted surgery (RVAS), and forty-eight focused on surgery without robotic/video assistance (WAS). Among these studies, six presented results for both groups. 

The meta-analysis showed that the highest worldwide prevalence was for the neck (WAS: 41% and RVAS: 45.3%), back (WAS: 37.7% and RVAS: 49.9%), lower back (WAS: 40.0% and RVAS: 37.8%), and shoulders (WAS: 27.3% and RVAS: 41.4%). Some studies reported a similar prevalence in the neck (WAS: [7,44] vs. RVAS: [7,48,77]), back (WAS: [61,89] vs. RVAS: [60,87]), lower back (WAS: [9,47] vs. RVAS: [71,82]), and shoulders (WAS: [11,84] vs. RVAS: [31,69]). These high prevalence rates have been reported in previous reviews or studies investigating other healthcare professionals [92]. Tavakkol et al. [93] identified a prevalence of 53.66% in the neck, 55.63% in the shoulders, and 61.48% in the lower back for nurses based on 12 studies. In physiotherapists, Gorce et al. [94] reported a prevalence of 26.4% in the neck, 40.1% in the lower back, and 20.8% in the shoulders from 26 studies. Rabiei et al. [95] and Kierklo et al. [6] estimated the prevalence in the neck (43.4% and 47%, respectively), back (35.8% and 35%, respectively) and shoulders (25% and 20%, respectively) of dentists. Okuyucu et al. [96] reported a similar WMSD prevalence in the neck (45.3%), lower back (71.4%), and shoulders (44.5%) among midwives. Among surgeons, Epstein et al. [15] summarized the prevalence of these three body areas, with a high prevalence in the neck (60%), shoulders (52%), and back (49%). The higher values for the neck and shoulders in comparison to the present work could be explained by the difference in sample size (77 vs. 21 studies) and by the fact that their study focused primarily on pathologies encountered by surgeons (degenerative cervical and lumbar spine disease and rotator cuff pathology). These prevalence rates suggest that health professionals, including surgeons, are exposed to high risks for these body areas. However, each profession has its own specificities (sociological, environmental, technical, ergonomic, etc.), which could explain the variations observed. In surgeons, prolonged static postures, forced exertion under improper conditions, occupational stress, a high precision level, time pressure, and high job demands are factors that increase the risk of WMSDs and their associated symptoms [97,98]. The CCOHS added general muscle fatigue, back pain, neck and shoulder stiffness, and other health problems to these conditions [99].

The use of equipment seems to affect the risk of prevalence for surgeons. The results showed that for the shoulders (WAS: 28.3% vs. RVAS: 41.9%), wrists (WAS: 20.9% vs. RVAS: 31.5%), and thumbs (WAS: 9.9% vs. RVAS: 21.8%), the prevalence was higher in RVAS (*p* < 0.05). This suggests that the use of material assistance could modify the postures and thus induce higher WMSD risks for the upper limbs. Special attention will, therefore, need to be paid to the surgeon’s position in relation to the patient and to the screens during surgical procedures. Rios et al. [100] investigated the positioning of different types of equipment and personnel in an operating room. Berguer et al. [3] compared surgeons’ postures and their variations during laparoscopy and open surgery. Kelts et al. [101] were interested in the positioning of screens in an operating room. The relative positioning of a health professional in relation to the patient, particularly in the context of lymphatic drainage, has been studied to optimize its use in order to reduce WMSD risks, particularly for the shoulders [102]. The ergonomics of the instruments is equally important. Hemal et al. [59] underlined the importance of studying the postural ergonomics of the hand in grip techniques during laparoscopy. 

Our results and the work mentioned above show the need to make surgeons aware of ergonomic problems, such as sitting at an optimal height in relation to the patient, having their eyes at the right height in relation to a screen, etc. In addition, it is necessary to be vigilant about controlling the causes that lead to the appearance of WMSDs. Awkward postures that are maintained for a long time due to inappropriate positioning relative to the patient can expose surgeons to risks of WMSDs in the neck, back, and upper limbs. To more easily identify these awkward postures, Jacquier-Bret et al. proposed the notion of a generic posture [103]. A non-optimal organization of all the elements/materials in the operating room could also explain these awkward postures.

These results are reinforced by the large number of studies included in the meta-analysis (77 studies). In comparison with previous studies, Epstein et al. [15] considered only 21 on surgeons, Tavakkol et al. [93] pooled 12 studies on nurses, and Gorce et al. [94] included 26 studies on physiotherapists. Despite this, a meta-analysis could not be conducted for three of the thirteen body areas studied (thumbs, mid-back, and hips) due to the insufficient number of studies (fewer than five). For the ten other body areas, the neck, shoulders, elbows, and wrists showed, for both the WAS and RVAS groups, a number of prevalence values higher than 15. The upper and lower back, knees, and ankles had a number of values higher than 20 for WAS but a lower number (<15) for RVAS. Finally, the back and fingers had a lower number of prevalence values for both groups of between 5 and 15.

However, in our meta-analysis, significant heterogeneity was observed (I^2^ > 80%) for both WAS and RVAS. This result reminds us that this parameter is essential in this type of study. Indeed, the size of the sample and the sensitivity of the tools used (type of questionnaires in particular) are major parameters that could largely explain this heterogeneity. In addition, the profile of the surgeons (age, sex, surgical experience, and experience with the use of different materials, particularly for RAVS), their geographical location (country and continent), and their working conditions (workload and specialty) are all elements to be monitored. Despite this heterogeneity, our analysis showed that one in five to one in two surgeons would be likely to develop WMSDs in half of the body areas studied, i.e., the neck, back, upper back, lower back, shoulders, and wrists. The use of a conservative random model reinforces the relevance of this result.

### 4.1. Limitations

Some limitations should be addressed. The first limitation concerns the method of data collection. The questionnaires used were significantly different in all studies. Some used tools presented in the literature, such as the Nordic musculoskeletal disorders questionnaire, while others used questionnaires specifically developed for the study. The understanding and interpretation of the questions may have led to variations in the assessment of the WMSD prevalence by body zone.

A second limitation may be the unrestricted inclusion of studies in the RVAS group. Indeed, the works prior to 2010 could have less advanced or even obsolete technologies that could distort the prevalence of the MSDs reported by the surgeons.

A third limitation concerns the variability in the sample sizes of surgeons in the included studies (17 to 1086). Although the meta-analyses weighted the results, it would be appropriate to reduce this difference to study the prevalence of MSDs.

A fourth limitation concerns the general objective of the study, i.e., to assess the general MSD prevalence by body area, which was conducted with high heterogeneity. No methods or sensitivity analyses were used to explore the possible causes of the heterogeneity among the studies or the robustness. We propose that sub-groups, e.g., the surgeons’ specialties (17 were considered in the meta-analysis), or a meta-regression could be used to investigate this heterogeneity (provided that sufficient work is available).

Another limitation concerns the PRISMA selection method. The selected inclusion criteria, i.e., limited to articles written in English or to the “original article” manuscript category, could have led to the exclusion or potential omission of works that could have completed and extended the results presented in this review and meta-analysis. 

### 4.2. Recommendations and Future Work

Considering the high prevalence of WMSDs, it seems even more important to increase surgeons’ awareness through ergonomic programs specific to their activities, with or without assistance. Future research is required to develop equipment and incorporate ergonomic features to prevent WMSDs during surgery. 

To overcome the problem of heterogeneity, it would be recommended to set up a more standardized protocol. An alternative would be to pool together studies with the same experimental conditions and similar workplaces (public vs. private), sex, experience, age, and all other factors that can affect the WMSD occurrence. 

Future work should be conducted (1) to propose innovative assistive devices and ergonomic adjustments to operating rooms and (2) for meta-analyses in order to increase the knowledge on the prevalence and reduce the risk of WMSDs among surgeons.

## 5. Conclusions

Surgeons are significantly exposed to WMSDs. The highest prevalence was found in the neck, back, lower back, and shoulders. The meta-analyses showed that RVAS increased the WMSD prevalence in the shoulders, wrist, and thumbs among surgeons. Future work could focus on work environment design, particularly on the positioning and adjustment of equipment, and on postural analysis to reduce the appearance of WMSDs. Methodological recommendations have been proposed to reduce the heterogeneity observed for future reviews and meta-analyses.

## Figures and Tables

**Table 1:** Quality appraisal of the 77 included studies according to the modified CONSORT 2010 checklist.

High Quality	Medium Quality		Low Quality
Giagio et al. [30]	Adams et al. [22]	Alnefaie et al. [31]	-
Mohseni-Bandpei et al. [10]	Alqahtani et al. [32]	Alqahtani et al. [12]	
	AlSabah et al. [33]	Alzahrani et al. [34]	
	Auerbach et al. [35]	Battevi et al. [36]	
	Berguer [37]	Bolduc-Beguin et al. [13]	
	Buschbacher [38]	Capone et al. [39]	
	Cass et al. [40]	Cavanagh et al. [41]	
	Cohen-Rosenblum et al. [42]	Dabholkar et al. [43]	
	Dabholkar et al. [44]	Davis et al. [45]	
	Davis et al. [46]	Dhimitri et al. [47]	
	Diaconita et al. [48]	Dianat et al. [14]	
	Esposito et al. [49]	Esser et al. [50]	
	Filisetti et al. [51]	Forst et al. [52]	
	Franasiak et al. [20]	Franasiak et al. [53]	
	Giberti et al. [54]	Gofrit et al. [55]	
	Goldstein et al. [56]	Grant et al. [57]	
	Hansel et al. [58]	Hemal et al. [59]	
	Ho et al. [60]	Hyer et al. [61]	
	Johnston et al. [62]	Kaya et al. [63]	
	Khansa et al. [8]	Kim-Fine et al. [64]	
	Kitzmann et al. [65]	Klein et al. [66]	
	Knudsen et al. [67]	Lee et al. [68]	
	Liang et al. [2]	Liang et al. [69]	
	Liberman et al. [25]	Mal and Costello, [70]	
	Matern and Koneczny [71]	Mavrovounis et al. [72]	
	Mehrifar et al. [73]	Miller et al. [74]	
	Mirbod et al. [75]	Morandeira-Rivas et al. [76]	
	O’Sullivan et al. [77]	Plerhoples et al. [7]	
	Quinn and Moohan [78]	Rambabu and Suneetha [79]	
	Rata et al. [80]	Riaz et al. [81]	
	Ruitenburg et al. [11]	Santos-Carreras et al. [82]	
	Sari et al. [83]	Sivak-Callcott et al. [84]	
	Stomberg et al. [85]	Szeto et al. [1]	
	Tan and Kwek [86]	Tjiam et al. [24]	
	Trejo et al. [87]	Vaghela et al. [88]	
	Vijendren et al. [89]	Voss et al. [90]	
	Wauben et al. [23]	Wohlauer et al. [9]	
	Wolf et al. [91]		

**Figure A1:**
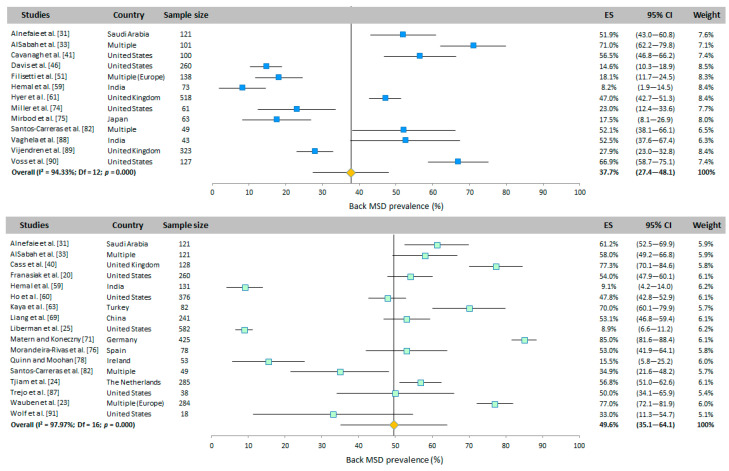
Meta-analysis of back WMSD prevalence for WAS (top panel in blue) and for RVAS (bottom panel in green). The orange diamond represents the overall prevalence for each group.

**Figure A2:**
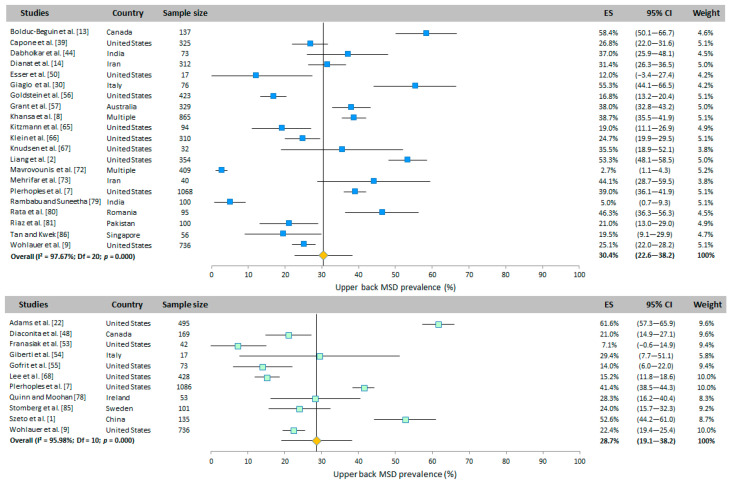
Meta-analysis of upper back WMSD prevalence for WAS (top panel in blue) and for RVAS (bottom panel in green). The orange diamond represents the overall prevalence for each group.

**Figure A3:**
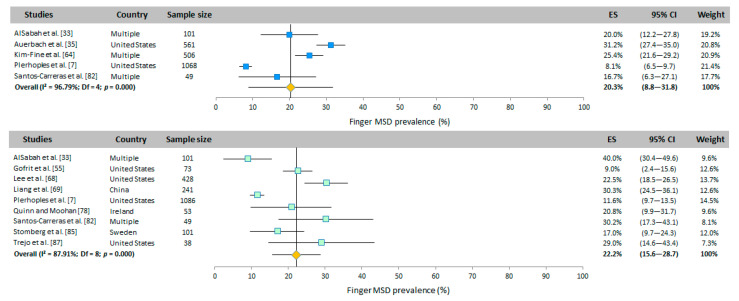
Meta-analysis of fingers WMSD prevalence for WAS (top panel in blue) and for RVAS (bottom panel in green). Orange diamond represents the overall prevalence for each group.

**Figure A4:**
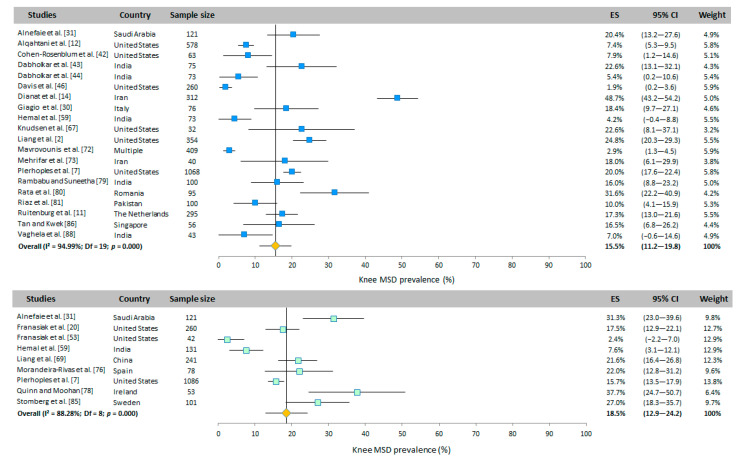
Meta-analysis of knee WMSD prevalence for WAS (top panel in blue) and for RVAS (bottom panel in green). Orange diamond represents the overall prevalence for each group.

**Figure A5:**
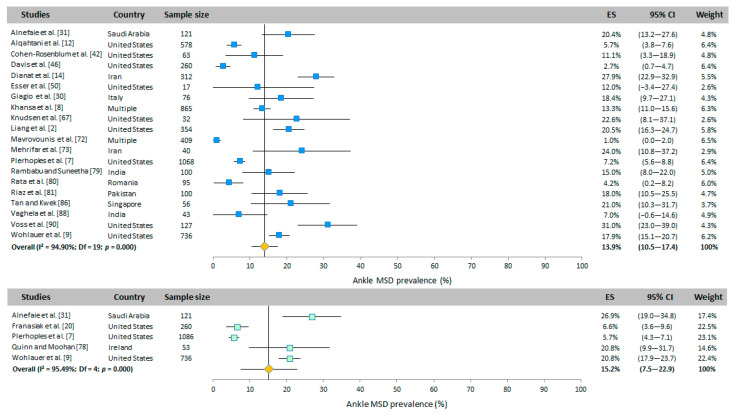
Meta-analysis of ankle WMSD prevalence for WAS (top panel in blue) and for RVAS (bottom panel in green). Orange diamond represents the overall prevalence for each group.

## Data Availability

Data available on request.

## Appendix A

### Appendix A.1

**Table A1:** Detailed quality appraisal of the 77 articles included in review using the modified CONSORT 2010 checklist [27].

		Introduction		Methods											Results						Discussion				Total	
	Title and Abstract	Background and Objective		Trial Design		Participants	Intervention	Outcomes		Sample Size		Randomization and Sequence Generation		Statistical Methods	Participant Flow	Baseline Data	Numbers Analyzed	Outcomes and Estimation	Ancillary Analyses	Harms	Limitations	Generalizability	Interpretation	Funding	Score/19	% Present Criteria
Authors	1	2A	2B	3A	3B	4	5	6A	6B	7A	7B	8A	8B	9	10	11	12	13	14	15	16	17	18	19		
Adams et al., 2013 [22]	1	1	1	0	-	1	1	1	-	0	-	0	-	1	1	1	1	1	1	-	1	0.5	0.5	1	14	74%
Alnifaie et al., 2019 [31]	1	1	1	0	-	1	1	1	-	0	-	0	-	1	1	1	1	0	0	-	0	0.5	0.5	0	11	58%
Alqahtani et al., 2015 [32]	1	1	1	0	-	1	1	1	-	0	-	0	-	1	1	1	1	0	0	-	1	0.5	0.5	0	12	63%
Alqhatani et al., 2022 [12]	1	1	1	0	-	1	1	1	-	0	-	0	-	1	1	1	1	0	0	-	1	0.5	0.5	0	12	63%
AlSabah et al., 2019 [33]	1	1	1	0	-	1	1	1	-	0	-	0	-	1	1	1	1	0	0	-	1	0	0.5	0	11.5	61%
Alzahrani et al., 2016 [34]	1	1	1	0	-	1	1	1	-	0	-	0	-	1	1	1	1	0	0	-	1	0.5	0.5	0	12	63%
Auerbach et al., 2011 [35]	1	1	1	0	-	1	1	1	-	0	-	0	-	1	1	1	1	0	0	-	0.5	1	1	0	12.5	66%
Battevi et al., 2009 [36]	1	1	1	0	-	1	1	1	-	0	-	0	-	1	1	1	1	0	0	-	0	0	0.5	0	10.5	55%
Berguer 1998 [37]	1	1	1	0	-	1	1	1	-	0	-	0	-	1	1	1	1	0	0	-	0	0	0.5	0	10.5	55%
Bolduc-Beguin et al., 2017 [13]	1	1	1	0	-	1	1	1	-	0	-	0	-	1	1	1	1	0	0	-	0	0	0.5	0	10.5	55%
Buschbacher 1994 [38]	1	1	1	0	-	1	1	1	-	0	-	0	-	1	1	1	1	0	0	-	0	0	0.5	0	10.5	55%
Capone et al., 2010 [39]	1	1	1	0	-	1	1	1	-	0	-	0	-	1	1	1	1	1	1	-	1	1	0.5	0.5	15	79%
Cass et al., 2014 [40]	1	1	1	0	-	1	1	1	-	0	-	0	-	1	1	1	1	0	0	-	0.5	0.5	0.5	0	11.5	61%
Cavanagh et al., 2012 [41]	1	1	1	0	-	1	1	1	-	0	-	0	-	1	1	1	1	0	0	-	1	0.5	1	0	12.5	66%
Cohen-Rosenblum et al., 2022 [42]	1	1	1	0	-	1	1	1	-	0	-	0	-	1	1	1	1	0	0	-	1	0.5	0.5	0	12	63%
Dabholkar et al., 2015 [43]	1	1	1	0	-	1	1	1	-	0	-	0	-	0	1	1	1	0	0	-	0	0.5	0.5	0	10	53%
Dabholkar et al., 2017 [44]	1	1	1	0	-	1	1	1	-	0	-	0	-	0	1	1	1	0	0	-	0	0	0.5	0.5	10	53%
Davis et al., 2013 [45]	1	1	1	0	-	1	1	1	-	0	-	0	-	1	1	1	1	0	0	-	0	0	0.5	0	10.5	55%
Davis et al., 2014 [46]	1	1	1	0	-	1	1	1	-	0	-	0	-	1	1	1	1	0	0	-	0	0	0.5	0	10.5	55%
Dhimitri et al., 2005 [47]	1	1	1	0	-	1	1	1	-	0	-	0	-	1	1	1	1	1	0	-	0	0	0.5	0	11.5	61%
Diaconita et al., 2019 [48]	1	1	1	0	-	1	1	1	-	0	-	0	-	0	1	1	1	0	0	-	0	0	0.5	0	9.5	50%
Dianat et al., 2018 [14]	1	1	1	0	-	1	1	1	-	0	-	0	-	1	1	1	1	1	1	-	1	1	1	0	15	79%
Esposito et al., 2014 [49]	1	1	1	0	-	1	1	1	-	0	-	0	-	1	1	1	1	0	0	-	0.5	0	0.5	0	11	58%
Esser et al., 2007 [50]	1	1	1	0	-	1	1	1	-	0	-	0	-	0	1	1	1	0	0	-	0	0.5	0.5	0	10	53%
Filisetti et al., 2015 [51]	1	1	1	0	-	1	1	1	-	0	-	0	-	0	1	1	1	0	0	-	0	0	0.5	0	9.5	50%
Forst et al., 2006 [52]	1	1	1	0	-	1	1	1	-	0	-	0	-	1	1	1	1	1	0	-	1	0.5	0.5	0	13	68%
Franasiak et al., 2012 [20]	1	1	1	0	-	1	1	1	-	0	-	0	-	1	1	1	1	0	0	-	1	0.5	0.5	0	12	63%
Franasiak et al., 2014 [53]	1	1	1	0	-	1	1	1	-	0	-	0	-	0	1	1	1	0	0	-	1	0	0.5	0	10.5	55%
Giagio et al., 2019 [30]	1	1	1	1	-	1	1	1	-	1	-	1	-	1	1	1	1	1	1	-	1	1	1	0	18	95%
Giberti et al., 2014 [54]	1	1	1	0	-	1	1	1	-	0	-	0	-	1	1	1	1	0	0	-	1	0.5	0.5	0	12	63%
Gofrit et al., 2008 [55]	1	1	1	0	-	1	1	1	-	0	-	0	-	1	1	1	1	0	0	-	0	0.5	0.5	0	11	58%
Goldstein et al., 2004 [56]	1	1	1	0	-	1	1	1	-	0	-	0	-	1	1	1	1	0	0	-	1	0.5	0.5	0	12	63%
Grant et al., 2020 [57]	1	1	1	0	-	1	1	1	-	0	-	0	-	1	1	1	1	1	0	-	1	0.5	0.5	0.5	13.5	71%
Hansel et al., 2008 [58]	1	1	1	0	-	1	1	1	-	0	-	0	-	1	1	1	1	1	1	-	1	0.5	0.5	0	14	74%
Hemal et al., 2001 [59]	1	1	1	0	-	1	1	1	-	0	-	0	-	1	1	1	1	0	0	-	0	0.5	0.5	0	11	58%
Ho et al., 2018 [60]	1	1	1	0	-	1	1	1	-	0	-	0	-	0.5	1	1	1	0	0	-	1	0.5	0.5	0	11.5	61%
Hyer et al., 2015 [61]	1	1	1	0	-	1	1	1	-	0	-	0	-	1	1	1	1	1	1	-	1	1	1	0	15	79%
Johnston et al., 2005 [62]	1	1	1	0	-	1	1	1	-	0	-	0	-	1	1	1	1	0	0	-	0.5	0.5	0.5	0	11.5	61%
Kaya et al., 2008 [63]	1	1	1	0	-	1	1	1	-	0	-	0	-	0.5	1	1	1	0	0	-	0	0	0.5	0	10	53%
Khansa et al., 2018 [8]	1	1	1	0	-	1	1	1	-	0	-	0	-	1	1	1	1	1	1	-	1	1	1	0	15	79%
Kim-Fine et al., 2013 [64]	1	1	1	0	-	1	1	1	-	0	-	0	-	1	1	1	1	1	1	-	0	0.5	0.5	0	13	68%
Kitzmann et al., 2012 [65]	1	1	1	0	-	1	1	1	-	0	-	0	-	1	1	1	1	1	1	-	1	1	1	0	15	79%
Klein et al., 2015 [66]	1	1	1	0	-	1	1	1	-	0	-	0	-	1	1	1	1	1	1	-	1	0.5	0.5	0	14	74%
Knudsen et al., 2014 [67]	1	1	1	0	-	1	1	1	-	0	-	0	-	1	1	1	1	0	0	-	1	0.5	0.5	1	12	63%
Lee et al., 2017 [68]	1	1	1	0	-	1	1	1	-	0	-	0	-	1	1	1	1	0	0	-	0.5	0.5	0.5	0	10.5	55%
Liang et al., 2012 [2]	1	1	1	0	-	1	1	1	-	0	-	0	-	1	1	1	0.5	0	0.5	-	1	0.5	0.5	0	10	53%
Liang et al., 2013 [69]	1	1	1	0	-	1	1	1	-	0	-	0	-	1	1	1	1	0	0	-	1	0.5	1	0	12	63%
Liberman et al., 2005 [25]	1	1	1	0	-	1	1	1	-	0	-	0	-	1	1	1	1	0	0	-	1	0.5	0.5	0	11	58%
Mal and Costello, 2002 [70]	1	1	1	0	-	1	1	1	-	0	-	0	-	0.5	1	1	1	0	0	-	0	0.5	0.5	0	9.5	50%
Matern and Koneczny 2007 [71]	1	1	1	0	-	1	1	1	-	0	-	0	-	0.5	1	1	1	0	0	-	0	0.5	0.5	0	9.5	50%
Mavrovounis et al., 2021 [72]	1	1	1	0	-	1	1	1	-	0	-	0	-	1	1	1	1	0	0	-	1	0.5	1	0.5	12.5	66%
Mehrifar et al., 2018 [73]	1	1	1	0	-	1	1	1	-	0	-	0	-	0.5	1	1	1	0	0	-	0	0.5	0.5	0	10	53%
Miller et al., 2012 [74]	1	1	1	0	-	1	1	1	-	0	-	0	-	1	1	1	1	1	0.5	-	0.5	0.5	0.5	0	12.5	66%
Mirbod et al. 1995 [75]	1	1	1	0	-	1	1	1	-	0	-	0	-	1	1	1	1	1	1	-	0	0.5	0.5	0	12.5	66%
Mohseni-Bandpei et al., 2011 [10]	1	1	1	0	-	1	1	1	-	0	-	0,5	-	1	1	1	1	1	1	-	1	1	1	1	16.5	87%
Morandeira-Rivas et al., 2012 [76]	1	1	1	0	-	1	1	1	-	0	-	0	-	1	1	1	1	0	0	-	1	0.5	0.5	0	12	63%
O’Sullivan 2002 [77]	1	1	1	0	-	1	1	1	-	0	-	0	-	1	1	1	1	0	0	-	0.5	0.5	0.5	0	11.5	61%
Plerhoples et al., 2012 [7]	1	1	1	0	-	1	1	1	-	0	-	0	-	1	1	1	1	0	0	-	0.5	0.5	0.5	0	11.5	61%
Quinn and Moohan 2015 [78]	1	1	1	0	-	1	1	1	-	0	-	0	-	1	1	1	1	0.5	0	-	0	0.5	0.5	0	11.5	61%
Rambabu and Suneetha 2014 [79]	1	1	1	0	-	1	1	1	-	0	-	0	-	1	1	1	1	0	0	-	1	0.5	0.5	0	12	63%
Rata et al., 2021 [80]	1	1	1	0	-	1	1	1	-	0	-	0	-	1	1	1	1	1	1	-	0.5	0.5	0.5	0.5	14	74%
Riaz et al., 2021 [81]	1	1	1	0	-	1	1	1	-	0	-	0	-	1	1	1	1	0	0	-	1	0.5	0.5	0	12	63%
Ruitenburg et al., 2012 [11]	1	1	1	0	-	1	1	1	-	0	-	0	-	1	1	1	1	0.5	0.5	-	0	0.5	0.5	0	12	63%
Santos-Carreras et al., 2012 [82]	1	1	1	0	-	1	1	1	-	0	-	0	-	1	1	1	1	0	0	-	1	1	1	1	14	74%
Sari et al., 2010 [83]	1	1	1	0	-	1	1	1	-	0	-	0	-	1	1	1	1	0	0	-	1	0.5	0.5	0.5	12.5	66%
Sivak-Callcott et al., 2011 [84]	1	1	1	0	-	1	1	1	-	0	-	0	-	1	1	1	1	0	0	-	0	0.5	0.5	0	11	58%
Stomberg et al., 2010 [85]	1	1	1	0	-	1	1	1	-	0	-	0	-	1	1	1	1	0	0	-	0	0.5	0.5	0	11	58%
Szeto et al., 2009 [1]	1	1	1	0	-	1	1	1	-	0	-	0	-	1	1	1	0.5	1	1	-	1	0.5	0.5	1	13	68%
Tan and Kwek 2020 [86]	1	1	1	0	-	1	1	1	-	0	-	0	-	1	1	1	1	0	0	-	0	0.5	0.5	0	10	53%
Tjiam et al., 2014 [24]	1	1	1	0	-	1	1	1	-	0	-	0	-	1	1	1	1	0	0	-	0	0.5	0.5	0	10	53%
Trejo et al., 2006 [87]	1	1	1	0	-	1	1	1	-	0	-	0	-	1	1	1	1	0	0	-	0	0.5	0.5	0	10	53%
Vaghela et al., 2019 [88]	1	1	1	0	-	1	1	1	-	0	-	0	-	1	1	1	1	0	0	-	0.5	0.5	0.5	0	10.5	55%
Vijendren et al., 2016 [89]	1	1	1	0	-	1	1	1	-	0	-	0	-	0.5	1	1	1	0	0	-	0	0	0.5	0	9.5	50%
Voss et al., 2016 [90]	1	1	1	0	-	1	1	1	-	0	-	0	-	1	1	1	1	1	1	-	1	0.5	0.5	0	13.5	71%
Wauben et al., 2006 [23]	1	1	1	0	-	1	1	1	-	0	-	0	-	1	1	1	1	0	0	-	0	0.5	0.5	0	10.5	55%
Wohlauer et al., 2021 [9]	1	1	1	0	-	1	1	1	-	0	-	0	-	1	1	1	1	1	1	-	0	0.5	0.5	0	12.5	66%
Wolf et al., 2000 [91]	1	1	1	0	-	1	1	1	-	0	-	0	-	1	1	1	1	0	0	-	0	0.5	0.5	0	10.5	55%

### Appendix A.2

**Table A2:** Characteristics of the 48 included WAS studies among surgeons. WMSD prevalence by body area was reported for each study (when available).

None Robotic-/Video-Assisted Surgery																	
Authors	Study Details				Body Area												
					Neck	Back	Upper Back	Mid-Back	Lower Back	Shoulder	Elbow	Wrist	Fingers	Thumb	Hip	Knee	Ankle
Alnefaie et al., 2019 [31]	**N Participants**	121	**Age (year)**	34.38	46.3%	51.9%				25.7%	9.3%	22.2%				20.4%	20.4%
	**Country**	Saudi Arabia	**Practice (year)**	NR													
	**Male/Female (%)**	61.2/38.8	**Case load (per week)**	NR													
Alqahtani et al., 2015 [32]	**N Participants**	183	**Age (year)**	54.9					27.9%	14.2%	14.2%	22.9%					
	**Country**	Multiple	**Practice (year)**	22.3													
	**Male/Female (%)**	97.8/2.2	**Case load (per week)**	5.8 cases													
Alqhatani et al., 2022 [12]	**N Participants**	578	**Age (year)**	52.8	18.3%				20.7%	14%	15.7%	31.3%			4%	7.4%	5.7%
	**Country**	United States	**Practice (year)**	19.78													
	**Male/Female (%)**	84.8/15.2	**Case load (per week)**	8.57 cases													
AlSabah et al., 2019 [33]	**N Participants**	101	**Age (year)**	45.2 ± 7.8	52%	71%				30%		12%	20%				
	**Country**	Multiple	**Practice (year)**	19.4 ± 7.5													
	**Male/Female (%)**	94.7/5.3	**Case load (per week)**	20.25 cases													
Alzahrani et al., 2016 [34]	**N Participants**	402	**Age (year)**	51.2	10.4%				28.6%	12.9%	15.4%	10%					
	**Country**	United States	**Practice (year)**	18.3													
	**Male/Female (%)**	76.1/23.9	**Case load (per week)**	NR													
Auerbach et al., 2011 [35]	**N Participants**	561	**Age (year)**	54	59.4%				62.2%	48.5%	28.3%	24.8%	31.2%				
	**Country**	United States	**Practice (year)**	NR													
	**Male/Female (%)**	NR	**Case load (per week)**	2.8 cases													
Bolduc-Beguin et al., 2017 [13]	**N Participants**	137	**Age (year)**	46	64.3%		58.4%		64.2%								
	**Country**	Canada	**Practice (year)**	14.0													
	**Male/Female (%)**	79.0/21.0	**Case load (per week)**	NR													
Capone et al., 2010 [39]	**N Participants**	325	**Age (year)**	48.1			26.8%	9.2%	24%	17.9%	29.8%	24.7%		12.6%			
	**Country**	United States	**Practice (year)**	12.9													
	**Male/Female (%)**	87.1/12.9	**Case load (per week)**	NR													
Cavanagh et al., 2012 [41]	**N Participants**	100	**Age (year)**	52.96 ± 8.0.3	59.7%	56.5%						19.4%					
	**Country**	United States	**Practice (year)**	21.17 ± 9.32													
	**Male/Female (%)**	85.0/15.0	**Case load (per week)**	NR													
Cohen-Rosenblum et al., 2022 [42]	**N Participants**	63	**Age (year)**	45.2	17.5%				30.2%	33.3%	11.1%	54%			9.5%	7.9%	11.1%
	**Country**	United States	**Practice (year)**	10.65													
	**Male/Female (%)**	0.0/100.0	**Case load (per week)**	5.3 cases													
Dabholkar et al., 2015 [43]	**N Participants**	75	**Age (year)**	43.4	26.6%				49.3%	14.6%	14.6%	25.3%				22.6%	
	**Country**	India	**Practice (year)**	14.06													
	**Male/Female (%)**	74.0/26.0	**Case load (per week)**	10.24 cases													
Dabholkar et al., 2017 [44]	**N Participants**	73	**Age (year)**	37.38 ± 10.79	41%		37%		32.9%	16.4%	9.6%	23.3%				5.4%	
	**Country**	India	**Practice (year)**	10.60 ± 9.1													
	**Male/Female (%)**	63.1/36.9	**Case load (per week)**	4.86 ± 2.23 cases													
Davis et al., 2013 [45]	**N Participants**	140	**Age (year)**	49.7 ± 10.7	10%				19.3%	7.1%		25%					
	**Country**	United States	**Practice (year)**	19.3													
	**Male/Female (%)**	NR	**Case load (per week)**	13.8 h													
Davis et al., 2014 [46]	**N Participants**	260	**Age (year)**	NR	7.7%	14.6%				3.5%		8.8%				1.9%	2.7%
	**Country**	United States	**Practice (year)**	20 ± 11													
	**Male/Female (%)**	80.0/20.0	**Case load (per week)**	19 ± 10 h													
Dhimitri et al., 2005 [47]	**N Participants**	697	**Age (year)**	51.9	32.6%				39%			32.9%					
	**Country**	United States	**Practice (year)**	19.8													
	**Male/Female (%)**	84.1/15.9	**Case load (per week)**	6.8 cases													
Dianat et al., 2018 [14]	**N Participants**	312	**Age (year)**	45.2 ± 9.3	45.8%		31.4%		42.3%	40.1%	13.8%	25%			28.5%	48.7%	27.9%
	**Country**	Iran	**Practice (year)**	12.5 ± 8.3													
	**Male/Female (%)**	65.1/34.9	**Case load (per week)**	13.1 ± 7.3 cases													
Esser et al., 2007 [50]	**N Participants**	17	**Age (year)**	39.6	59%		12%	12%	41%	53%		12%					12%
	**Country**	United States	**Practice (year)**	6.8													
	**Male/Female (%)**	70.6/29.4	**Case load (per week)**	24.3 h													
Filisetti et al., 2015 [51]	**N Participants**	138	**Age (year)**	NR		18.1%											
	**Country**	Multiple (Europe)	**Practice (year)**	15													
	**Male/Female (%)**	76.1/23.9	**Case load (per week)**	1.4 case													
Forst et al., 2006 [52]	**N Participants**	285	**Age (year)**	46.7								28.8%					
	**Country**	United States	**Practice (year)**	14.1													
	**Male/Female (%)**	97.5/2.5	**Case load (per week)**	NR													
Giagio et al., 2019 [30]	**N Participants**	76	**Age (year)**	37.7 ± 12.1	78.9%		55.3%		71.1%	51.3%	3.9%	26.3%			14.5%	18.4%	18.4%
	**Country**	Italy	**Practice (year)**	10.4 ± 11.3													
	**Male/Female (%)**	65.8/34.2	**Case load (per week)**	16.1 cases													
Goldstein et al., 2004 [56]	**N Participants**	423	**Age (year)**	NR			16.8%		29.4%								
	**Country**	United States	**Practice (year)**	13.8													
	**Male/Female (%)**	NR	**Case load (per week)**	9.8 cases													
Grant et al., 2020 [57]	**N Participants**	329	**Age (year)**	NR	59%		38%			55%							
	**Country**	Australia	**Practice (year)**	NR													
	**Male/Female (%)**	73.0/27.0	**Case load (per week)**	20 h													
Hemal et al., 2001 [59]	**N Participants**	73	**Age (year)**	36.9 ± 8.4	6%	8.2%				10.2%	5.4%	8.6%				4.2%	
	**Country**	India	**Practice (year)**	10.9 ± 7.6													
	**Male/Female (%)**	NR	**Case load (per week)**	12.9 ± 5.7 h													
Hyer et al., 2015 [61]	**N Participants**	518	**Age (year)**	48.7	29.6%	47%											
	**Country**	United Kingdom	**Practice (year)**	NR													
	**Male/Female (%)**	74.5/25.5	**Case load (per week)**	8.78 h													
Khansa et al., 2018 [8]	**N Participants**	865	**Age (year)**	NR	66.6%		38.7%	16.2%	39.9%	52%		38.3%					13.3%
	**Country**	Multiple	**Practice (year)**	NR													
	**Male/Female (%)**	75.8/24.2	**Case load (per week)**	7 cases													
Kim-Fine et al., 2013 [64]	**N Participants**	506	**Age (year)**	41.9	50.3%			43%	53.9%	38.6%	17%	15.6%	25.4%				
	**Country**	Multiple	**Practice (year)**	15.9													
	**Male/Female (%)**	49.0/51.0	**Case load (per week)**	NR													
Kitzmann et al., 2012 [65]	**N Participants**	94	**Age (year)**	41.5 ± 10.9	46%		19%		26%	11%	1%	17%					
	**Country**	United States	**Practice (year)**	11.8													
	**Male/Female (%)**	66.0/34.0	**Case load (per week)**	NR													
Klein et al., 2015 [66]	**N Participants**	310	**Age (year)**	49			24.7%		34.4%								
	**Country**	United States	**Practice (year)**	16													
	**Male/Female (%)**	89.0/11.0	**Case load (per week)**	11.1 cases													
Knudsen et al., 2014 [67]	**N Participants**	32	**Age (year)**	29.5	59.4%		35.5%		54.8%	34.4%	3.1%	19.4%			9.7%	22.6%	22.6%
	**Country**	United States	**Practice (year)**	2.9													
	**Male/Female (%)**	75.0/25.0	**Case load (per week)**	33.8 h													
Liang et al., 2012 [2]	**N Participants**	354	**Age (year)**	44.5	65.2%		53.3%		63.1%	61.5%	13.8%	36.9%				24.8%	20.5%
	**Country**	United States	**Practice (year)**	8.9													
	**Male/Female (%)**	70.9/29.1	**Case load (per week)**	NR													
Mal and Costello 2002 [70]	**N Participants**	367	**Age (year)**	51.2						24%							
	**Country**	United Kingdom	**Practice (year)**	NR													
	**Male/Female (%)**	NR	**Case load (per week)**	NR													
Mavrovounis et al., 2021 [72]	**N Participants**	409	**Age (year)**	44.63	4.6%		2.7%		6.8%	4.2%	1.7%	2%				2.9%	1%
	**Country**	Multiple	**Practice (year)**	15													
	**Male/Female (%)**	82.4/17.6	**Case load (per week)**	4 cases													
Mehrifar et al., 2015 [73]	**N Participants**	40	**Age (year)**	38.54 ± 8.34	84.6%		44.1%		56.8%	37.4%	10%	40%			20%	18%	24%
	**Country**	Iran	**Practice (year)**	8.7													
	**Male/Female (%)**	63.0/37.0	**Case load (per week)**	NR													
Miller et al., 2012 [74]	**N Participants**	61	**Age (year)**	50 ± 9.5		23%											
	**Country**	United States	**Practice (year)**	16.16.0													
	**Male/Female (%)**	79.0/21.0	**Case load (per week)**	NR													
Mirbod et al. 1995 [75]	**N Participants**	63	**Age (year)**	41.8 ± 9.5	20.6%	17.5%			36.5%	17.5%		9.5%					
	**Country**	Japan	**Practice (year)**	16.6 ± 9.5													
	**Male/Female (%)**	100.0/0.0	**Case load (per week)**	NR													
Mohseni-Bandpei et al., 2011 [10]	**N Participants**	223	**Age (year)**	42.6					71.7%								
	**Country**	Iran	**Practice (year)**	10.5													
	**Male/Female (%)**	48.4/51.6	**Case load (per week)**	NR													
Plerhoples et al., 2012 [7]	**N Participants**	1068	**Age (year)**	45.8 ± 9.0	46.6%		39%		51.8%	12.3%	4.6%	9.3%	8.1%	7.2%	11.4%	20%	7.2%
	**Country**	United States	**Practice (year)**	13.8 ± 9.2													
	**Male/Female (%)**	72.7/27.3	**Case load (per week)**	0.77 case													
Rambabu and Suneetha 2014 [79]	**N Participants**	100	**Age (year)**	NR	11%		5%		20%	8%	5%	8%			12%	16%	15%
	**Country**	India	**Practice (year)**	NR													
	**Male/Female (%)**	NR	**Case load (per week)**	NR													
Rata et al., 2021 [80]	**N Participants**	95	**Age (year)**	37.56 ± 8.74	55.8%		46.3%		74.7%	46.3%	17.7%	16.8%			11.6%	31.6%	4.2%
	**Country**	Romania	**Practice (year)**	10.09 ± 8.41													
	**Male/Female (%)**	62.1/37.9	**Case load (per week)**	7.56 ± 3.73 cases													
Riaz et al., 2021 [81]	**N Participants**	100	**Age (year)**	33.13± 11	37%		21%		45%	35%	5%	22%			9%	10%	18%
	**Country**	Pakistan	**Practice (year)**	7.48± 9.51													
	**Male/Female (%)**	48.0/52.0	**Case load (per week)**	24.78 cases													
Ruitenburg et al., 2012 [11]	**N Participants**	295	**Age (year)**	40.0 ± 9.8	30.2%				23.4%	25.8%						17.3%	
	**Country**	The Netherlands	**Practice (year)**	NR													
	**Male/Female (%)**	45.0/55.0	**Case load (per week)**	NR													
Santos-Carreras et al., 2012 [82]	**N Participants**	49	**Age (year)**	43 ± 8.36	52.1%	52.1%				20.8%		12.5%	16.7%				
	**Country**	Multiple	**Practice (year)**	NR													
	**Male/Female (%)**	87.8/12.2	**Case load (per week)**	NR													
Sivak-Callcott et al., 2011 [84]	**N Participants**	130	**Age (year)**	48	58%			29.8%	31.3%	26.7%							
	**Country**	United States	**Practice (year)**	16.1													
	**Male/Female (%)**	85.4/14.6	**Case load (per week)**	13.8 h													
Tan and Kwek 2020 [86]	**N Participants**	56	**Age (year)**	33	66.1%		19.5%		37%	39.3%	8.5%	42.9%			5%	16.5%	21%
	**Country**	Singapore	**Practice (year)**	NR													
	**Male/Female (%)**	NR	**Case load (per week)**	16h													
Vaghela et al., 2019 [88]	**N Participants**	43	**Age (year)**	42.07	30.2%	52.5%				23.3%	2.3%	2.3%			2.3%	7%	7%
	**Country**	India	**Practice (year)**	15.14													
	**Male/Female (%)**	69.8/30.2	**Case load (per week)**	NR													
Vijendren et al., 2016 [89]	**N Participants**	323	**Age (year)**	NR	29.7%	27.9%						9%					
	**Country**	United Kingdom	**Practice (year)**	18.7													
	**Male/Female (%)**	NR	**Case load (per week)**	NR													
Voss et al., 2016 [90]	**N Participants**	127	**Age (year)**	NR	65.4%	66.9%				54.2%		30%					31%
	**Country**	United States	**Practice (year)**	7.1													
	**Male/Female (%)**	60.6/39.4	**Case load (per week)**	NR													
Wohlauer et al., 2021 [9]	**N Participants**	736	**Age (year)**	51.4 ± 10.9	45.2%		25.1%		39%			6.3%					17.9%
	**Country**	United States	**Practice (year)**	17.2 ± 11.6													
	**Male/Female (%)**	83.6/16.4	**Case load (per week)**	NR													

### Appendix A.3

**Table A3:** Characteristics of the 35 included RVAS studies among surgeons. WMSD prevalence by body area was reported for each study (when available).

Robotic-/Video-Assisted Surgery																	
Authors	Study Details				Body Area												
					Neck	Back	Upper Back	Mid-Back	Lower Back	Shoulder	Elbow	Wrist	Fingers	Thumb	Hip	Knee	Ankle
Adams et al., 2013 [22]	**N Participants**	495	**Age (year)**	47	72.9%		61.6%		75.6%	66.6%		60.9%					
	**Country**	United States	**Practice (year)**	18													
	**Male/Female (%)**	50.3/49.7	**Case load (per week)**	8 h													
Alnefaie et al., 2019 [31]	**N Participants**	121	**Age (year)**	34.38	49.3%	61.2%				35.8%	13.4%	29.9%				31.3%	26.9%
	**Country**	Saudi Arabia	**Practice (year)**	NR													
	**Male/Female (%)**	61.2/38.8	**Case load (per week)**	NR													
AlSabah et al., 2019 [33]	**N Participants**	101	**Age (year)**	45.2 ± 7.8	39%	58%				58%		40%	40%				
	**Country**	Multiple	**Practice (year)**	19.4 ± 7.5													
	**Male/Female (%)**	94.7/5.3	**Case load (per week)**	20.25 cases													
Battevi et al., 2009 [36]	**N Participants**	176	**Age (year)**	42.8						34.3%	11.8%						
	**Country**	Italy	**Practice (year)**	NR													
	**Male/Female (%)**	38.6/61.4	**Case load (per week)**	NR													
Berguer 1998 [37]	**N Participants**	149	**Age (year)**	NR	52%					55%		47%					
	**Country**	United States	**Practice (year)**	NR													
	**Male/Female (%)**	NR	**Case load (per week)**	NR													
Buschbacher 1994 [38]	**N Participants**	265	**Age (year)**	48.8 ± 8.6	13%					19%	15%	14%					
	**Country**	United States	**Practice (year)**	NR													
	**Male/Female (%)**	95.1/4.9	**Case load (per week)**	NR													
Cass et al., 2014 [40]	**N Participants**	128	**Age (year)**	NR	73.4%	77.3%				80.5%		69.5%					
	**Country**	United Kingdom	**Practice (year)**	NR													
	**Male/Female (%)**	NR	**Case load (per week)**	NR													
Diaconita et al., 2019 [48]	**N Participants**	169	**Age (year)**	NR	46%		21%		36%	28%		18%					
	**Country**	Canada	**Practice (year)**	NR													
	**Male/Female (%)**	68.0/32.0	**Case load (per week)**	NR													
Esposito et al., 2014 [49]	**N Participants**	23	**Age (year)**	NR						43.5%	4.3%	21.7%					
	**Country**	Multiple	**Practice (year)**	9.78													
	**Male/Female (%)**	NR	**Case load (per week)**	7.28 cases													
Franasiak et al., 2012 [20]	**N Participants**	260	**Age (year)**	45.3	58.8%	54%				53.6%	26.1%	44.1%				17.5%	6.6%
	**Country**	United States	**Practice (year)**	9.4													
	**Male/Female (%)**	59.2/40.8	**Case load (per week)**	NR													
Franasiak et al., 2014 [53]	**N Participants**	42	**Age (year)**	NR	33.3%		7.1%		19.1%	23.8%		16.7%			2.4%	2.4%	
	**Country**	United States	**Practice (year)**	3.0													
	**Male/Female (%)**	45.2/54.8	**Case load (per week)**	5.4 h													
Giberti et al., 2014 [54]	**N Participants**	17	**Age (year)**	51.3			29.4%	11.8%	5.9%								
	**Country**	Italy	**Practice (year)**	3.0													
	**Male/Female (%)**	94.1/5.9	**Case load (per week)**	6.0 h													
Gofrit et al., 2008 [55]	**N Participants**	73	**Age (year)**	44 ± 8.4	19%		14%		19%	17.5%	9%	10.5%	9%				
	**Country**	United States	**Practice (year)**	11.7 ± 8.4													
	**Male/Female (%)**	100.0/0.0	**Case load (per week)**	3.1 ± 2.8 h													
Hansel et al., 2008 [58]	**N Participants**	71	**Age (year)**	45.1	10.4%							16.7%					
	**Country**	United States	**Practice (year)**	NR													
	**Male/Female (%)**	83.1/16.9	**Case load (per week)**	NR													
Hemal et al., 2001 [59]	**N Participants**	131	**Age (year)**	41.6 ± 6.1	13%	9.1%				18%	15%	16%				7.6%	
	**Country**	India	**Practice (year)**	4.8 ± 2.5													
	**Male/Female (%)**	NR	**Case load (per week)**	8.8 ± 4.8 h													
Ho et al., 2018 [60]	**N Participants**	376	**Age (year)**	34.7 ± 8.69	73.5%	47.8%				52.8%							
	**Country**	United States	**Practice (year)**	4.48													
	**Male/Female (%)**	72.7/27.3	**Case load (per week)**	6.7 cases													
Johnston et al., 2005 [62]	**N Participants**	25	**Age (year)**	NR	69%				68%	55%	48%	54%					
	**Country**	United States	**Practice (year)**	NR													
	**Male/Female (%)**	NR	**Case load (per week)**	1.6 case													
Kaya et al., 2008 [63]	**N Participants**	82	**Age (year)**	NR	72%	70%											
	**Country**	Turkey	**Practice (year)**	NR													
	**Male/Female (%)**	NR	**Case load (per week)**	NR													
Lee et al., 2017 [68]	**N Participants**	428	**Age (year)**	47.61 ± 9.2	21.4%		15.2%		12.3%	9.5%		9.4%	22.5%				
	**Country**	United States	**Practice (year)**	13.48 ± 9.5													
	**Male/Female (%)**	71.3/28.7	**Case load (per week)**	5.24 ± 4.2 cases													
Liang et al., 2013 [69]	**N Participants**	241	**Age (year)**	44.3	58.1%	53.1%				33.6%		32%	30.3%			21.6%	
	**Country**	China	**Practice (year)**	NR													
	**Male/Female (%)**	96.7/3.3	**Case load (per week)**	5.28 cases													
Liberman et al., 2005 [25]	**N Participants**	582	**Age (year)**	48	11.2%	8.9%						44.2%					
	**Country**	United States	**Practice (year)**	14.8													
	**Male/Female (%)**	89.3/10.7	**Case load (per week)**	17 cases													
Matern and Koneczny 2007 [71]	**N Participants**	425	**Age (year)**	NR	60%	85%				39%							
	**Country**	Germany	**Practice (year)**	NR													
	**Male/Female (%)**	75.0/25.0	**Case load (per week)**	NR													
Morandeira-Rivas et al., 2012 [76]	**N Participants**	78	**Age (year)**	44.06	55%	53%				52%		50%				22%	
	**Country**	Spain	**Practice (year)**	NR													
	**Male/Female (%)**	87.0/13.0	**Case load (per week)**	1.3 case													
O’Sullivan 2002 [77]	**N Participants**	122	**Age (year)**	NR	46%					16%	8%	36%					
	**Country**	Canada	**Practice (year)**	NR													
	**Male/Female (%)**	NR	**Case load (per week)**	NR													
Plerhoples et al., 2012 [7]	**N Participants**	1086	**Age (year)**	45.8 ± 9.0	44.1%		41.4%		44.5%	33.2%	8.5%	16.6%	11.6%	17.8%	10.1%	15.7%	5.7%
	**Country**	United States	**Practice (year)**	12.1 ± 8.3													
	**Male/Female (%)**	72.7/27.3	**Case load (per week)**	0.77 case													
Quinn and Moohan 2015 [78]	**N Participants**	53	**Age (year)**	30.4	41.5%	15.5%	28.3%		28.3%	43.4%	15.1%	20.8%	20.8%	22.6%		37.7%	20.8%
	**Country**	Ireland	**Practice (year)**	4.5													
	**Male/Female (%)**	36.0/64.0	**Case load (per week)**	NR													
Santos-Carreras et al., 2012 [82]	**N Participants**	49	**Age (year)**	43 ± 8.36	37.2%	34.9%				41.9%		20.9%	30.2%				
	**Country**	Multiple	**Practice (year)**	NR													
	**Male/Female (%)**	87.8/12.2	**Case load (per week)**	NR													
Sari et al., 2010 [83]	**N Participants**	55	**Age (year)**	39.5 ± 7.3	15%				26%	45%							
	**Country**	The Netherlands	**Practice (year)**	8.75 ± 6.07													
	**Male/Female (%)**	65.4/34.6	**Case load (per week)**	6 h													
Stomberg et al., 2010 [85]	**N Participants**	101	**Age (year)**	48.2 ± 10.2	50%		24%		55%	51%	6%	14%	17%			27%	
	**Country**	Sweden	**Practice (year)**	14.5 ± 10.4													
	**Male/Female (%)**	36.6/63.4	**Case load (per week)**	1.9 h													
Szeto et al., 2009 [1]	**N Participants**	135	**Age (year)**	35.3	82.9%		52.6%		68.1%	57.8%							
	**Country**	China	**Practice (year)**	10.0 ± 7.3													
	**Male/Female (%)**	82.2/17.8	**Case load (per week)**	NR													
Tjiam et al., 2014 [24]	**N Participants**	285	**Age (year)**	46	59.3%	56.8%				51.2%	26%	21.4%		24.9%			
	**Country**	The Netherlands	**Practice (year)**	12.9													
	**Male/Female (%)**	93.0/7.0	**Case load (per week)**	6.45 h													
Trejo et al., 2006 [87]	**N Participants**	38	**Age (year)**	NR	65%	50%				48%		55%	29%				
	**Country**	United States	**Practice (year)**	NR													
	**Male/Female (%)**	NR	**Case load (per week)**	NR													
Wauben et al., 2006 [23]	**N Participants**	284	**Age (year)**	45 ± 9	78%	77%				77%							
	**Country**	Multiple (Europe)	**Practice (year)**	NR													
	**Male/Female (%)**	89.4/10.6	**Case load (per week)**	17h													
Wohlauer et al., 2021 [9]	**N Participants**	736	**Age (year)**	51.4 ± 10.9	24%		22.4%		44.2%			4.9%					20.8%
	**Country**	United States	**Practice (year)**	17.2 ± 11.6													
	**Male/Female (%)**	83.6/16.4	**Case load (per week)**	NR													
Wolf et al., 2000 [91]	**N Participants**	18	**Age (year)**	45.5	28%	33%				17%	11%	67%					
	**Country**	United States	**Practice (year)**	NR													
	**Male/Female (%)**	NR	**Case load (per week)**	NR													
